# Supplementary material for: Sequestration within peptide coacervates improves the fluorescence intensity, kinetics, and limits of detection of dye-based DNA biosensors
Source: Commun Chem. 2024 Feb 29;7:49. doi: 10.1038/s42004-024-01124-3 (PMC10904739; doi:10.1038/s42004-024-01124-3)
Supplement: Supplementary file 2 — Supplementary Information [file 42004_2024_1124_MOESM2_ESM.pdf]

Supplementary Information: Sequestration within Peptide Coacervates Improves the Fluorescence Intensity, Kinetics, and Limits of Detection of Dye-based DNA Biosensors

Christopher M. Green[1,†], Deborah Sementa[2,†], Divita Mathur[3], Joseph S. Melinger[4], Priyasha Deshpande[5], Shana Elbaum-Garfinkle[5,6], Igor L. Medintz[1], Rein V. Ulijn[2,6,7], Sebastián A. Díaz[1,*]

† These authors contributed equally

1. Center for Bio/Molecular Science and Engineering Code 6900, U.S. Naval Research Laboratory, Washington, D.C. 20375, USA. sebastian.diaz@nrl.navy.mil

2. Nanoscience Initiative at Advanced Science Research Center, Graduate Center of the City University of New York, New York, NY 10031, USA.

3. Department of Chemistry, Case Western Reserve University, Cleveland, OH 44106, USA.

4. Electronics Sciences and Technology Division, U.S. Naval Research Laboratory, Washington, DC 20375, USA

5. Structural Biology Initiative at Advanced Science Research Center, Graduate Center of the City University of New York, New York, NY 10031, USA.

6. Ph.D. Programs in Biochemistry and Chemistry, The Graduate Center of the City University of New York, New York, New York 10016, USA.

7. Department of Chemistry Hunter College, City University of New York, New York, New York 10065, USA.

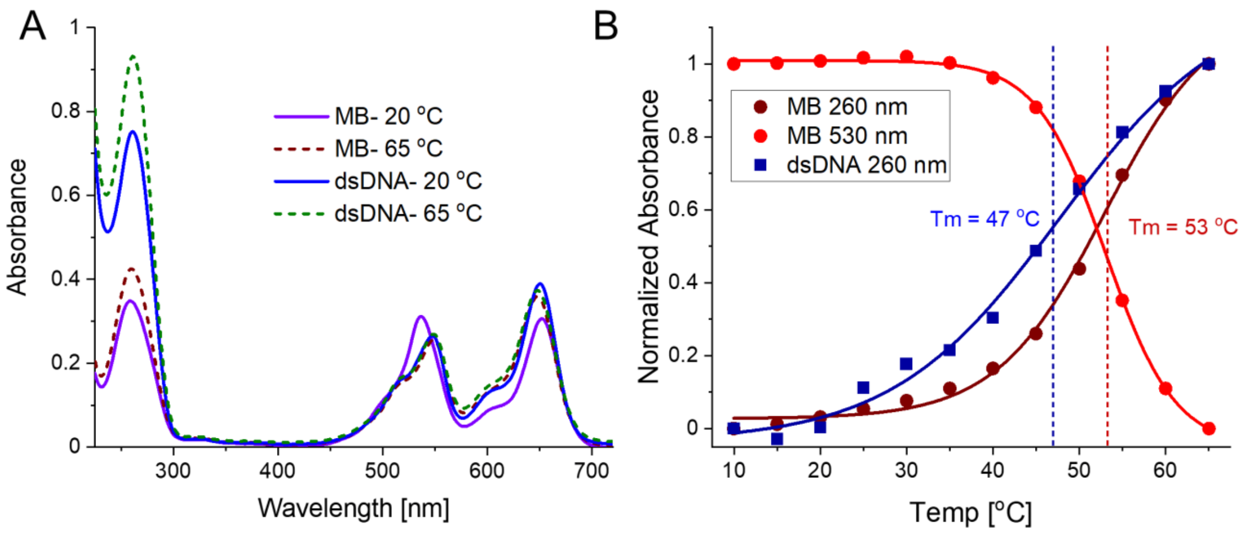

Figure S1: A) Absorbance spectra of the MB DNA, and a 2:1 target:MB samples (dsDNA) in buffer at 20 and 65 °C. B) Normalized absorbance of the 260 nm and or 530 nm absorbance of the MB and dsDNA samples. The 260 nm peak increases as the DNA goes from ds to ss, while the 530 nm peak decreases as the vibronic coupling of the Cy3-Cy5 due to their close-proximity diminishes upon heating. Curves are Boltzmann fits, and melting temperatures are determined at by the fitting inflection point.

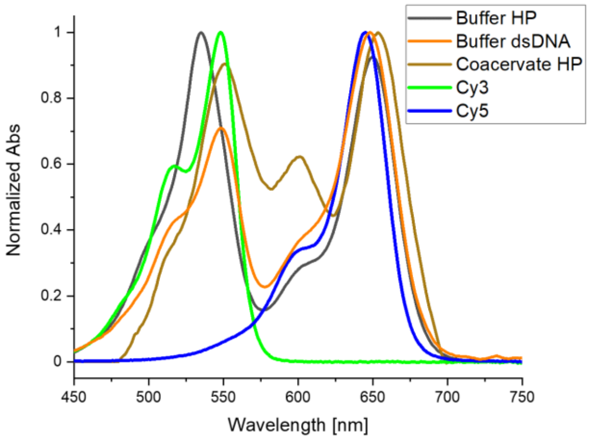

Figure S2: Normalized absorbance spectra of Cy3, Cy5, and FRET pair in buffer conditions, as well as the FRET pair in Coacervate conditions. It can be observed that neither FRET pair is the linear combination of the individual dyes. This suggests that the dyes are strongly coupled, which has often been shown to lead to non-radiative decay pathways for both the donor (Cy3) and acceptor (Cy5) dye.

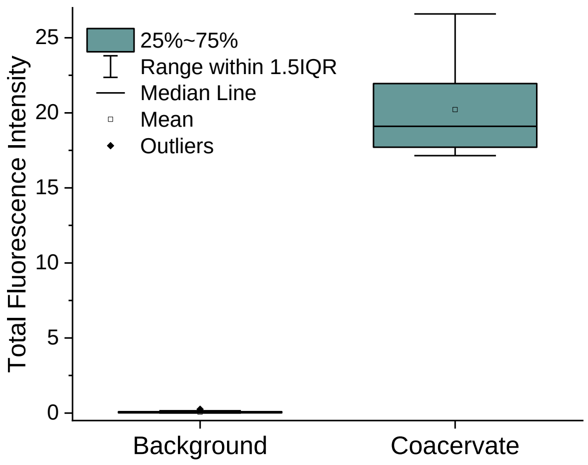

Figure S3: Representative data from fluorescence microscopy (Coacervates with 1:2 MB:target addition) showing the total fluorescence within the coacervates and the background. Data collected from 2 different images and 10 region of interests (ROIs). Average values are 0.06 ± 0.08 counts for background and 20.2 ± 3.3 counts for coacervates, this results in a value of 99.7 ± 0.4 % of fluorescence within coacervates.

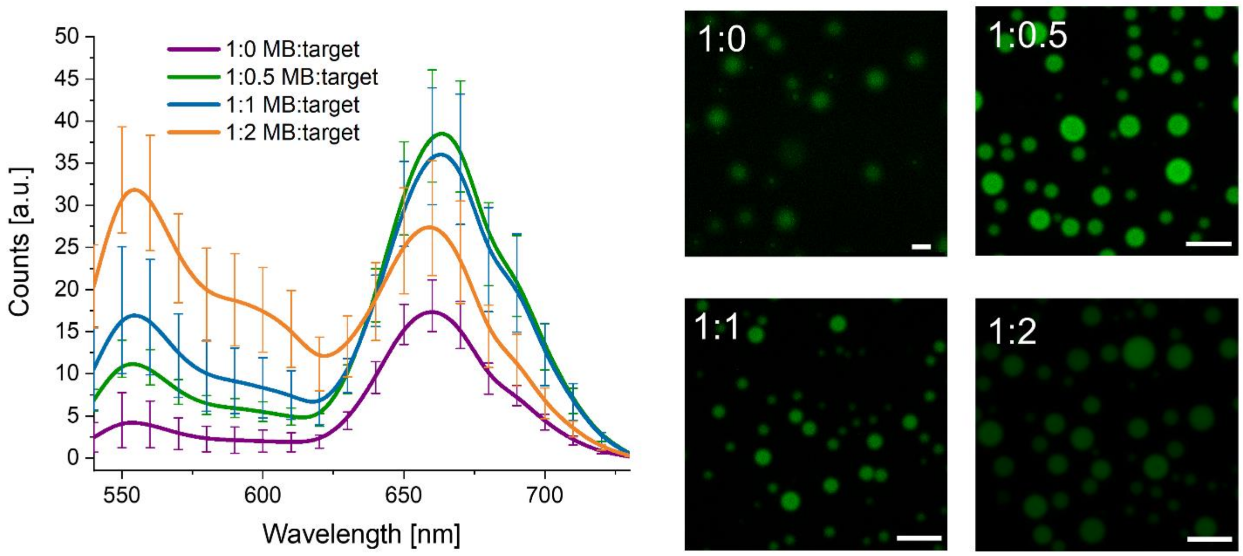

Figure S4: Fluorescence spectra obtained from confocal fluorescence microscopy of coacervates with pre-formed MB to target ratios. Images on the right are representative of the data on the left. Scale bar is 5 µm. Uncertainties arise from averaging the spectra of over 10 different images and ROI for each preparation.

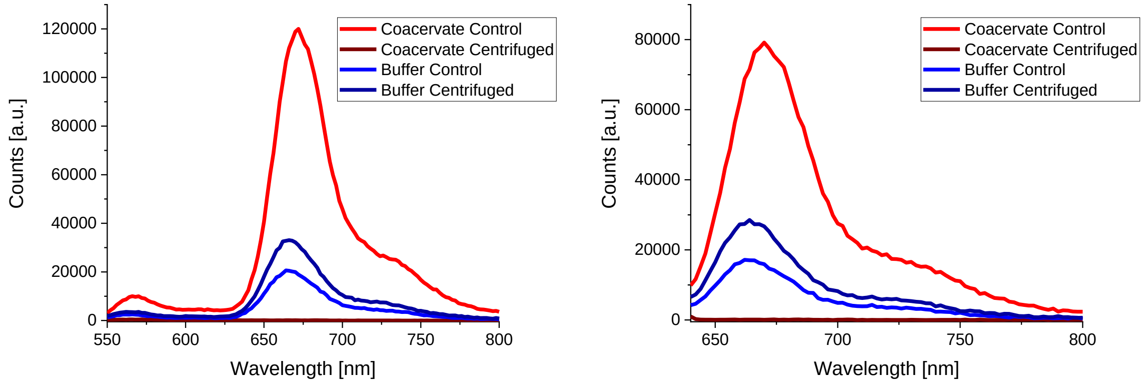

Figure S5: Confirmation of sequestration of DNA within coacervates. (Left) HP form of DNA, excite 530 nm. (Right) Cy5-DNA excite 615 nm. 250 nM of dye labeled DNA was added to either the Buffer solution or a Coacervate solution. After 20 minutes both samples were split into two samples and one was centrifuged at 10 min at 15k rpm and the fluorescence of the supernatant (Centrifuged) was compared to the non-centrifuged (Control) sample. As can be seen the buffer sample actually had slightly higher fluorescence after centrifugation, while the remaining fluorescence in the coacervate centrifugation samples was less than 0.3 ± 0.1%.

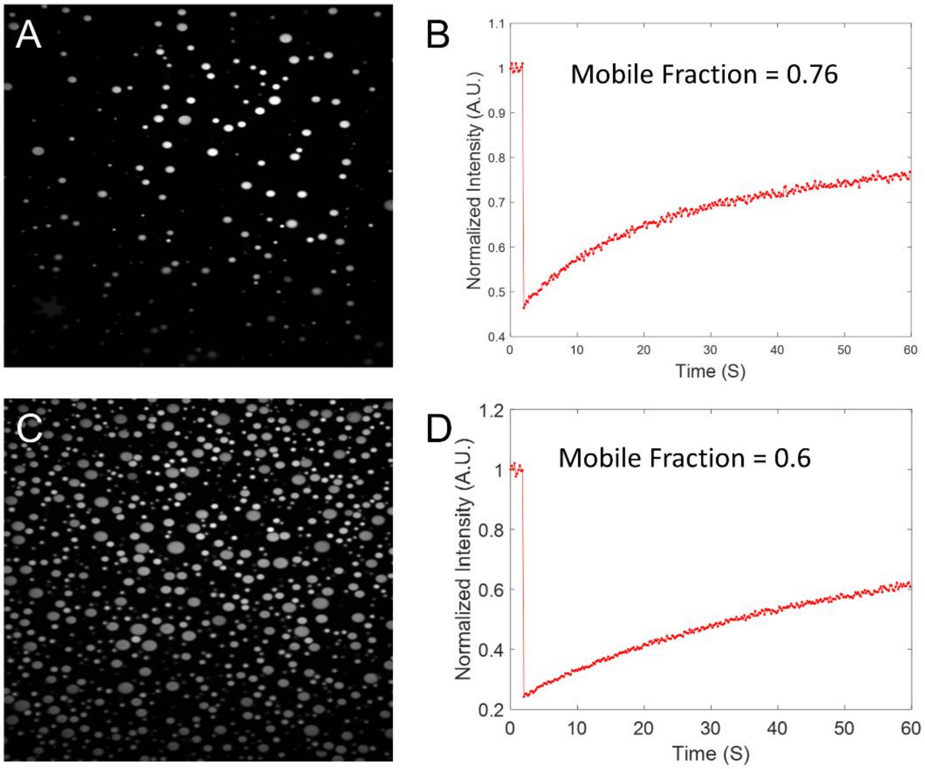

Figure S6: FRAP experiments as detailed in the Methods section in main text. A) Microscopy image of coacervates with MB in HP form. B) Representative FRAP curve from sample in (A). C) Microscopy image of coacervates with MB+target in dsDNA form. D) Representative FRAP curve from sample in (C). The experiment qualitatively demonstrates that the coacervates are dynamic and that the DNA and peptides are capable of moving within the structures. Quantitative estimates are determined by the Mobile Fraction as determined by the fitting process detailed below. We observe that the HP form appears to be slightly more mobile than the dsDNA, which is reasonable considering it has a lower molecular weight and is more compact.

The recovery of fluorescence was normalized using the double-normalization equation –

$$I\ (t)DoubleNormal = \left[\frac{(1/_{npre})\sum_{t=1}^{npre} I(t)ROI2'}{I(t)ROI2'}\right] \times \left[\frac{I(t)ROI1'}{(1/_{npre})\sum_{t=1}^{npre} I(t)ROI1'}\right] \qquad \text{Eq. S1}$$

Where *npre* = number of frames pre-bleaching, I(t) ROI1' and I(t) ROI2' are the fluorescence intensities of the bleached spot and the unbleached spot within the condensate respectively upon background subtraction. Normalized data is plotted as a function of time and fitted using exponential function of the form per EasyFRAP analysis manual to extract mobile fraction (mf)

Ifit = I0 - a.e(-β.t) – g e(-δ.t) → Mf = $\frac{a+\gamma}{1-(I0-a-\gamma)}$ Eq. S2

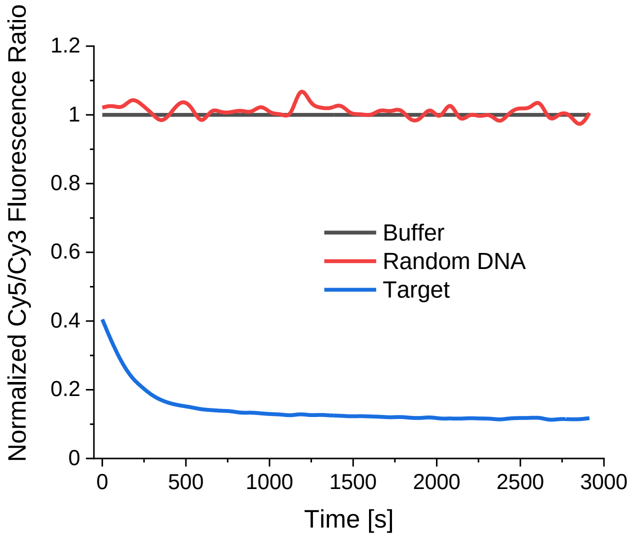

Figure S7: Control demonstrating random DNA sequence (ATATAATCGCTCGCATATTATGACTG) does not activate MB fluorescence signal change. Target and Random DNA added at 500 nM.

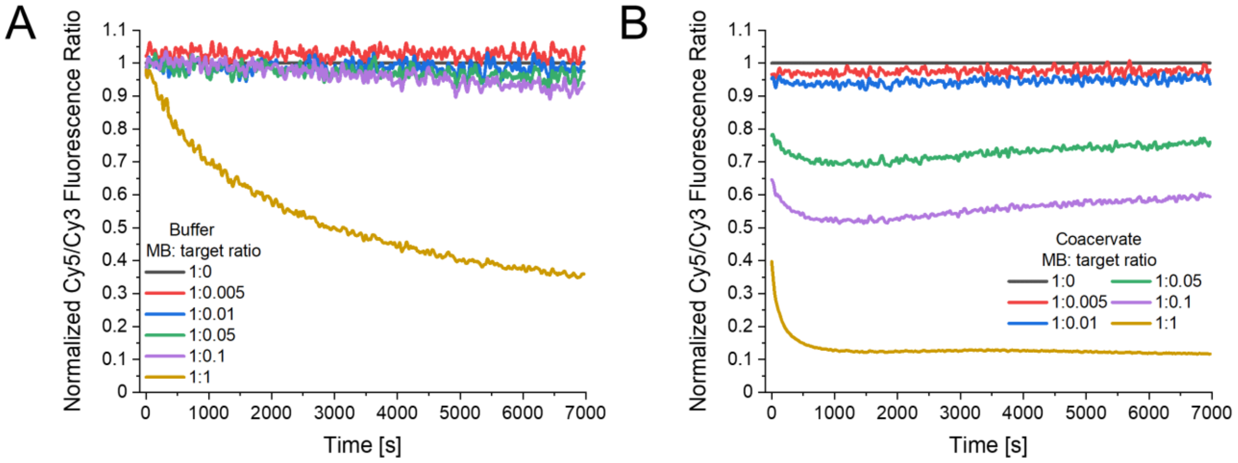

Figure S8: Representative kinetics of MB reacting to target strand addition in (A) buffer or (B) coacervates. The data set shown here is the average of two runs and was used, along with other similar experiments, to create the LOD Figure 6 in the main text.

Supplementary Note 1: Description of Förster resonance energy transfer system:

The following primer elucidates the discussion realized in the main text on the MB characterization; for a deeper reading, we recommend recent reviews that include detailed discussions on Förster resonance energy transfer (FRET).[1-2]

For a single donor (D) and acceptor (A) pair undergoing FRET, the efficiency of the transfer (E_{FRET}) can be experimentally determined by either the change on fluorescence lifetime (τ) or steady-state fluorescence intensity (I) of the donor.

$$E_{FRET} = 1 - \frac{\tau_{DA}}{\tau_D} = 1 - \frac{I_{DA}}{I_D} \qquad \text{Eq. S1}$$

Where the subscripts $_{\mathrm{D}}$ and $_{\mathrm{DA}}$ denote the presence of the D alone or D and A together, for our case we would consider the control MB-strand with only Cy3 as the D and the MB as the DA system. The two MB conformation, HP and dsDNA, result in distinct E_{FRET} values. The value of E_{FRET} determinations is that they can be correlated to distance between the D-A pair (r_{DA}) through spectroscopic characterization. The Förster distance, R_0, also referred to as the Förster radius, correlates to the separation distance at which D–A pair has $E_{\mathrm{FRET}} = 0.50$. The R_0 is a measure of the quality of a particular D–A pair, generally ranging from 2 to 10 nm, and can be determined by knowing the photophysical properties of the D-A dyes.

$$(R_0)^6 = 0.02108 \frac{\kappa^2 J Q_D}{n^4} \qquad \text{Eq. S2}$$

In equation S2, κ^2 is the dipole orientation factor, Q_{D} is the D fluorescence quantum yield, and n is the medium's refractive index. J is the overlap integral, a measure of the overlap of the D fluorescence emission and the A absorbance, and can be determined using:

$$J = \int_0^{\infty} F_D(\lambda) \varepsilon_A(\lambda) \lambda^4 d\lambda \qquad \text{Eq. S3}$$

where $F_{\mathrm{D}}(\lambda)$ is the D emission spectrum normalized to 1, $\varepsilon_{\mathrm{A}}(\lambda)$ is the A extinction coefficient in units of $\mathrm{M}^{-1}\ \mathrm{cm}^{-1}$, and λ is the wavelength in nm. With the experimental E_{FRET} and having the calculated R_0 we can determine the r_{DA} through the following equation:

$$E_{FRET} = \frac{1}{1+(r_{DA}/R_0)^6} \qquad \text{Eq. S4}$$

Supplementary References

1. Algar, W. R.; Hildebrandt, N.; Vogel, S. S.; Medintz, I. L., FRET as a Biomolecular Research Tool — Understanding Its Potential While Avoiding Pitfalls. *Nat. Methods* **2019,** *16*, 815-829.
2. Mathur, D.; Díaz, S. A.; Hildebrandt, N.; Pensack, R. D.; Yurke, B.; Biaggne, A.; Li, L.; Melinger, J. S.; Ancona, M. G.; Knowlton, W. B.; Medintz, I. L., Pursuing Excitonic Energy Transfer with Programmable DNA-Based Optical Breadboards. *Chem. Soc. Rev.* **2023**.
