# Supplementary material for: Sequestration within peptide coacervates improves the fluorescence intensity, kinetics, and limits of detection of dye-based DNA biosensors
Source: Commun Chem. 2024 Feb 29;7:49. doi: 10.1038/s42004-024-01124-3 (PMC10904739; doi:10.1038/s42004-024-01124-3)
Supplement: Supplementary file 3 — Description of Additional Supplementary Files [file 42004_2024_1124_MOESM3_ESM.pdf]

Description of Additional Supplementary Files

File name: Supplementary Data 1

Description: Data presented in figures in excel format. Each tab correlates to a figure, panels are separated within each tab.
